# Sofosbuvir and Simeprevir for the Treatment of Recurrent Hepatitis C with Fibrosing Cholestatic Hepatitis after Liver Transplantation

**Published:** 2016-02-01

**Authors:** D. Issa, B. Eghtesad, N. N. Zein, L. Yerian, M. Cruise, N. Alkhouri, R. Adams, I. A. Hanouneh

**Affiliations:** 1 Department of Gastroenterology and Hepatology, Cleveland Clinic, Cleveland, Ohio, United States,; 2 Department of General Surgery, Transplant Center, Cleveland Clinic, Cleveland, Ohio, United States,; 3 *Department of Surgical Pathology, Cleveland Clinic, Cleveland, Ohio, United States*

**Keywords:** Jaundice, obstructive, Hepacivirus, Liver transplantation, Liver cirrhosis, Fibrosis, Pancreatitis, Pruritus

## Abstract

Fibrosing cholestatic hepatitis (FCH) is an aggressive form of hepatitis C virus (HCV) recurrence after orthotopic liver transplantation (OLT), which frequently results in graft failure and death. Treatment of FCH remains challenging, and the optimal antiviral therapy is yet to be determined. Between November 2013 and early 2015, 62 patients with HCV cirrhosis underwent OLT at our transplant center, of whom, 5 patients developed recurrence HCV in the form of severe FCH and were treated with sofosbuvir and simeprevir (SOF-SMV) for 24 weeks. All patients achieved significant improvement of HCV viral load and had undetectable viral PCR at 6–8 week of treatment. The HCV RNA remained undetectable throughout treatment course. The first two patients achieved SVR at week 12 after completion of the treatment. There were significant histologic and biomarkers improvements after initiation of the treatment. One patient developed refractory pruritus and acute pancreatitis. The second, fourth and fifth patients had very benign treatment courses with no side effects recorded. The third patient was starting the treatment with multiple comorbid conditions. His course was complicated with hepatic artery thrombosis, and later developed sepsis and renal failure. Therefore, it seems that the combination of SOF-SMV is an efficacious oral regimen in OLT recipient with recurrent hepatitis C and FCH. However, safety profile needs to be carefully evaluated.

## INTRODUCTION

Hepatitis C virus (HCV) infection is a leading cause of cirrhosis and liver cancer and remains a primary indication for orthotopic liver transplantation (OLT) in the Western World [1]. Among OLT recipients, those with chronic HCV have a significantly lower 5-year survival rate in comparison with other recipients; the reason behind this is the inevitable recurrence of HCV after transplantation [2, 3]. Reinfection with HCV after OLT can follow different patterns; one of the most aggressive patterns is known as fibrosing cholestatic hepatitis (FCH). This form of HCV recurrence in the allograft represents a serious complication and affects 5%–14% of OLT performed for HCV [4, 5]. Most FCH cases are fatal secondary to rapidly progressive liver dysfunction followed by graft loss, which is usually seen within the first 1–2 years after OLT [6]. Only a few cases have been reported in the literature on successful treatment of post-transplantation FCH [7-10].

Traditional treatment with pegylated interferon and ribavirin combination has been attempted in selected OLT recipients with severe HCV recurrence but this use is limited by frequent adverse effects and low efficacy [11, 12]. Oral direct antiviral agents (DAA) such as telaprevir and boceprevir were approved by the US Food and Drug Administration (FDA) in 2011 [13-15], but their use post-OLT is also limited due to the significant drug-to-drug interactions particularly with immunosuppressant agents used post-OLT, such as cyclosporine, tacrolimus and sirolimus.

In late 2013 and early 2014, a major step was made in the treatment of HCV with the introduction of two new oral agents—sofosbuvir and simeprevir. Sofosbuvir is a nucleotide analogue inhibitor of the HCV NS5B RNA-dependent polymerase, whereas simeprevir is a specific inhibitor of the HCV NS3/4A serine protease [16-18]. Both are administered orally once daily. In this article, we report our experience with the use of interferon-free regimen for the treatment of HCV recurrence with severe FCH post-OLT. Our regimen included daily sofosbuvir (SOF) 400 mg plus simeprevir (SMV) 150 mg. The duration of treatment was determined to be 24 weeks as all of the patients were complicated or had multiple co-morbidities.

## CASES PRESENTATION

Between November 2013 and early 2015, 62 patients with HCV cirrhosis underwent OLT at the Cleveland Clinic, of whom, five patients developed recurrence of HCV post-OLT in the form of severe FCH. All five patients were treated with the regimen of sofosbuvir and simeprevir (SOF-SMV) for 24 weeks. All liver biopsies were reviewed by two expert pathologists, and the diagnosis of FCH was made according to definition proposed by the International Liver Transplantation Society.

Case 1

A 68-year-old white female presented with end-stage liver disease due to chronic HCV genotype 1b infection (Table 1). HCV was likely contracted after a blood transfusion approximately 30 years before. Prior to transplantation, the patient failed previous treatment with regular interferon and ribavirin in 1999 for HCV infection. Her liver disease progressed over years and was complicated by jaundice, refractory ascites and esophageal varices. At the time of transplantation, she had a laboratory Models for End-Stage Liver Disease (MELD) score of 40. Post-operatively, the patient received triple immunosuppressive therapy consisted of tacrolimus, mycophenolate mofetil (MMF) and prednisone. Prednisone was discontinued on post-operative day 21 while remained on tacrolimus and MMF with complete normalized liver function tests.

**Table 1:** Baseline characteristics of patients

Case	Age	Sex	Race	IM*	Genotype	MELD^†^ at OLT^‡^
1	68	Female	White	Tac^¶^, MMF^§^, steroids	1b	40
2	56	Female	White	Tac, MMF, steroids	1a	22
3	62	Male	African American	Tac, MMF, steroids	1a	25
4	44	Male	White	Tac, MMF, steroids	1a	31
5	30	Female	White	Tac, MMF, steroids	1a	37


**Figure 1:**
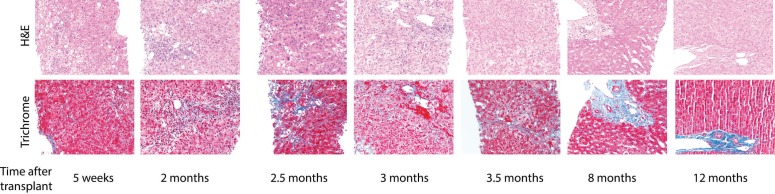
Photomicrograph of liver (Case 1) in post-transplantation period

Two months following OLT, the patient presented with fatigue and worsening jaundice. Liver chemistry tests were noted to be markedly abnormal with rise in the total bilirubin (TB) levels to 16.2 mg/dL, aspartate aminotransferase (AST) to 530 U/L, alanine aminotransferase (ALT) to 284 U/L, alkaline phosphate (ALP) to 1467 U/L, and gamma-glutamyl transpeptidase (GGT) to 749 U/L. Vascular ultrasound imaging of the liver showed patent hepatic vasculature and no intra- or extra-hepatic biliary duct dilation. A liver biopsy was performed and showed marked lobular and portal inflammation with hepatocyte swelling, focal cholestasis and patchy bile duct injury. Importantly, there was no portal or central vein endotheliitis. No significant fibrosis was seen on the trichrome stain (Fig 1). This histologic features were concerning for evolving recurrent cholestatic hepatitis C. The patient’s HCV viral load prior to the biopsy was 40,500,000 RNA IU/mL. Given the clinical and pathologic findings the decision was made to start anti-viral therapy with SOF-SMV.

Shortly after initiating anti-viral therapy, liver biochemical tests started to drift down (Table 2). However, three weeks later the liver enzymes went-up again and her TB reached a level of 52.2 mg/dL; the patient was feeling more fatigue. A repeated liver biopsy showed plasma cell-rich, immune-mediated hepatitis with prominent bile duct injury, favoring acute cellular rejection *vs*. plasma cell hepatitis. During that event, tacrolimus level was noted to be on the lower side of 2–4 ng/mL. The dose of tacrolimus was increased accordingly and the patient was started on high doses of intravenous steroid, followed by prednisone tapering. The patient responded well to the treatment and immunosuppression was continued with prednisone, tacrolimus and MMF. Anti-viral therapy was continued without interruption throughout the course. Tacrolimus trough level remained stable (8–10 ng/mL) during the rest of the treatment course and no dose changes were made.

**Table 2:** HCV RNA viral load and liver function tests prior to and after treatment with SOF-SMV

Patient 1										
Time	Viral load	T. Bili		AST		ALT		ALP		GGT
Pre-Rx	40,500,000	16.2		530		284		1467		749
Week 4	596	40.8		477		155		744		426
Week 12	undetectable	6.5		174		105		590		503
Week 24	undetectable	0.9		49		33		544		413
SVR 12	undetectable	0.7		59		56		449		546
Patient 2										
Time	Viral load	T. Bili		AST		ALT		ALP		GGT
Pre-Rx	9,120,000	5.0		353		85		253		194
Week 4	< 43	0.6		26		19		97		30
Week 12	undetectable	0.4		17		13		119		19
Week 24	undetectable	0.3		23		14		110		26
SVR 12	undetectable	0.4		16		20		115		28
Patient 3										
Time	Viral load	T. Bili		AST		ALT		ALP		GGT
Pre-Rx	69,000,000	21.8		192		82		125		314
Week 2	3200	19.6		57		28		122		104
Week 6	undetectable	6.7		62		26		92		43
Patient 4										
Time	Viral load		T. Bili		AST		ALT		ALP	GGT
Pre-Rx	8,970,000		10.8		415		259		244	296
Week 4	44		7.6		791		521		156	171
Week 12	N/A		2.9		42		49		180	144
Week 24	undetectable		1.0		35		36		78	98
SVR 12	undetectable		0.9		32		31		72	86
Patient 5										
Time	Viral load		T. Bili		AST		ALT		ALP	GGT
Pre-Rx	1,280,000		2.4		204		55		231	841
Week 4	9520		5.2		14		13		189	73
Week 12	undetectable		0.5		15		6		131	12
Week 24	undetectable		0.7		18		12		109	32
SVR 12	undetectable		0.7		21		15		78	25

The HCV viral load decreased to 596 IU/mL at week four of the therapy, then became undetectable at week 12, and remained undetectable to at least 12 weeks after completion of the treatment. The patient had a delayed but remarkable biochemical improvement. The course of the treatment was complicated by mild episode of acute pancreatitis that responded well to intravenous hydration and conservative management. The etiology of acute pancreatitis remained indeterminate. The treatment was also complicated by pruritus, which was refractory to medical therapy, and the patient underwent frequent sessions of plasmapheresis to relieve her symptoms. After completing the treatment, the patient’s pruritus resolved and plasmapheresis was no longer needed. 

The liver biopsy performed 12 weeks after the completion of SOF-SMV demonstrates near normal histology with no evidence of ongoing hepatic injury. There was no evidence of acute or chronic rejection, no chronic portal or lobular inflammation. The bile ducts were normal without evidence of chronic cellular rejection. Additionally, there was no residual fibrosis seen on the trichrome stain. 

Case 2

A 56-year-old female with HCV genotype 1a infection who previously failed treatment with interferon monotherapy in 1996 presented to our clinic for further management. After the diagnosis of a 2-cm hepatocellular carcinoma (HCC), she received a MELD upgrade to 22 points and received a liver from a cardiac-dead donor.

The post-transplantation immunosuppression regimen consisted of tacrolimus, MMF and prednisone. Prednisone was discontinued on post-operative day 21 while MMF was stopped six months following OLT. The patient remained on tacrolimus monotherapy six months post-OLT. 

Six months post-OLT, a routine protocol liver biopsy showed mild lobular inflammation and mild steatosis, suggestive of early recurrent HCV. The patient remained asymptomatic until nine months post-OLT. Her liver chemistry gradually trended up. Ultrasonography of the liver was normal with no lesions. No intra- or extra-hepatic biliary duct dilation was seen. A repeated liver biopsy demonstrated diffuse hepatocyte swelling and cholestasis. The trichrome stain demonstrates periportal fibrosis with focal pericellular and perisinusoidal fibrosis. The findings were compatible with fibrosing cholestatic recurrence of HCV. The HCV viral load at this time was 9,120,000 IU/mL. 

We decided to initiate treatment with SOF-SMV. Tacrolimus dosage needed to be increased to keep the level in the range of 4–6 ng/mL. HCV viral load improved to <43 IU/mL at week four, then became undetectable at week eight and remained undetectable at week 12 and at the end of the treatment. The patient had a complete normalization of liver enzymes at week two of the treatment. Her liver chemistry continued to be within normal limits and the patient has completed 24 weeks of therapy. She has experienced no adverse events. The patient achieved SVR with undetectable HCV RNA at 12 weeks after treatment completion. An additional liver biopsy performed three months after initiation of the therapy, showed resolution of the cholestatic changes and hepatocellular injury with only minimal inflammation without significant peri-sinusoidal fibrosis. Comparison of the patient’s previous liver biopsy, prior to SOF-SMV, demonstrated a significant histologic improvement with anti-viral therapy.

Case 3

The third patient was a 62-year-old male with multiple medical problems included HCV genotype 1a, coronary artery disease, hypertension and type 2 diabetes mellitus. He developed liver cirrhosis and HCC secondary to HCV infection. With exceptional MELD points granted for HCC the patient received a liver from a brain-dead donor. Prior to transplantation, the patient failed treatment with pegylated interferon and ribavirin.

After two months of transplantation, the patient had worsening jaundice and was frequently complaining of abdominal pain. AST and ALT were elevated and serum TB was reached 22.1 mg/dL. The patient underwent ERCP that showed a focal biliary stricture in the post-transplantation anastomosis. A liver biopsy at this time demonstrated focal mild portal and lobular inflammation with mild ductular reactive without changes of cellular rejection. Additionally there was no definite hepatocellular injury or fibrosis. Biliary sphincterotomy with dilatation and stenting of the anastomotic stricture were performed but did not alleviate patient’s symptoms. He continued to have a worsening jaundice despite a repeated ERCP and successfully replacing the stent. A liver biopsy performed four months post-OLT revealed diffuse hepatocyte swelling with associated hepatocyte injury, and no evidence of endotheliitis. The trichrome stain confirmed the presence of periportal and perilobular fibrosis with rare fibrous bridge. These features were consistent with fibrosing cholestatic hepatitis C. His HCV viral load was >69,000,000 IU/mL.

Treatment for HCV was started with SOF-SMV. HCV viral load decreased to 3200 IU/mL at week two and became undetectable at week six of the treatment. His liver biochemistries improved markedly at week six of the treatment. However, the patient developed recurrent abdominal pain, nausea and vomiting that required two ER visits. Liver ultrasonography was concerning for hepatic artery stenosis. Hepatic angiogram showed marked stenosis suggesting transplanted hepatic artery thrombosis. Paracentesis was performed for new ascites and fluid analysis showed severe leukocytosis and cultures later grew *E. coli*. He went into respiratory failure, required endotracheal intubation and stayed in the ICU for three days. He died of sepsis and multiorgan failure, which complicated his ICU stay.

Case 4

The fourth patient was a 44-year-old male with chronic infection with hepatitis B virus (HBV) and history of HCV-HIV co-infection. With a MELD score of 31, the patient received OLT from a matched 68-year-old donor. Post-OLT immunosuppression included tacrolimus, MMF and steroids. Prior to transplantation, his CD4 count was 194 and HBV DNA was undetectable. His antiviral therapy for HIV and HBV were continued post-OLT. This included emcitrabine 200 mg daily, tenofovir 300 mg daily, etravirin 200 mg twice daily, and raltegravir 400 mg twice daily. His liver enzymes were slowly trending up at 4.5 months post-OLT. A liver biopsy showed lobular architecture that was distorted by cholestatic changes with ballooning hepatocytes, lobular inflammation, and multiple acidophilic bodies. Portal tracts demonstrated moderate chronic inflammation with focal interface activity. There was only focal mild bile ductal injury. The trichrome stain highlighted mild portal expansion. These findings were consistent with HCV recurrence with FCH. Treatment for FCH was started with SOF-SMV; his HIV and HBV therapy were continued. 

HCV RNA viral load was 8,970,000 IU/mL prior to SOF-SMV treatment. It improved to 44 IU/mL at week four of the treatment. His liver chemistry improved slowly throughout the treatment course. At week 12 of the treatment, his TB decreased to 2.9 mg/dL, AST to 42 U/L, ALT to 49 U/L, ALP to 180 U/L, and GGT to 144 U/L. The patient completed 24 weeks and his HCV viral load remained undetectable. HCV viral load was also checked at week 12 after completion of the treatment and the patient successfully achieved SVR. No side effects were reported throughout the course of the treatment. 

Case 5

The fifth patient was a 30-year-old female with a history of α_1_ antitrypsin deficiency for which she underwent OLT in 1987. Her course was complicated with HCV genotype 1a infection that was likely acquired at the time of OLT. She developed cirrhosis post-OLT secondary to HCV infection and subsequently received a second liver transplantation in June 2014. Post-OLT immunosuppression included tacrolimus, MMF and prednisone. The last drug was discontinued on post-operative day 21. 

Two months after the second transplantation, the patient was admitted to the hospital with elevated liver enzymes and abdominal pain. Abdominal imaging was unremarkable. Liver biopsy showed evidence of recurrent HCV in the form of FCH. There were marked diffuse hepatocyte swelling with cholestasis, mild steatosis and scattered lobular inflammatory cell infiltrates. There was no portal vein endotheliitis, and bile duct damage was minimal. Trichrome stain demonstrated portal fibrous expansion with focal pericellular fibrosis. She was subsequently started on SOF-SMV therapy for HCV infection. HCV RNA prior to the treatment viral load of 1,280,000 IU/mL decreased to 59 IU/mL at week eight of the treatment, then became undetectable at week 12 of the treatment. Liver function tests prior to the treatment were as following: TB of 2.4 U/L, AST of 204 U/L, ALT of 55 U/L, ALP of 231 U/L, and GGT of 841 U/L. After two weeks of the treatment, her TB trended up to 5.2 U/L; however, all of her other liver functions improved remarkably; AST decreased to 14 U/L, ALT to 13 U/L, ALP to 189 U/L, and GGT to 73 U/L. The patient’s bilirubin dropped thereafter and reached normal limits at week six of the treatment. She had a complete normalization of her liver tests at week eight, and this was maintained at week 12 and week 24 of the treatment. The patient completed 24 weeks of therapy with no significant adverse events; she achieved SVR with undetectable HCV viral load 12 weeks after completion of the treatment. 

## DISCUSSION

HCV infection recurs in virtually all patients following OLT. The natural history of post-OLT recurrent HCV is different from that prior to transplantation [3]. Within few months post-OLT, 25%–45% of patients develop histological changes consistent with recurrent HCV; after few years, 80%–100% of patients progress to chronic hepatitis; 5%–10% of HCV patients recur in a severe and aggressive form known as FCH. Despite the new therapies available for the treatment of chronic HCV and the current golden era in antiviral pharmacology, recurrence of HCV after OLT remains a big treatment challenge. Furthermore, when HCV recurs in the form of FCH, the prognosis becomes much worse and aggressive treatment is not only indicated but also life-saving.

FCH might develop early after OLT. In fact, there are cases reported the development of FCH within one month of OLT [19]. On average, FCH is diagnosed after 7.6 months of transplantation [5]. Higher HCV viral load, earlier recurrence of HCV infection and relatively higher immunosuppressant levels are associated with the development of FCH [20]. Pegylated interferon and ribavirin use is poorly tolerated in OLT patients and has limited efficacy [10]. Moreover, telaprevir and boceprevir are not recommended in patients post-OLT due to high-risk interaction with immunosuppressive medications [21].

Herein, we report five cases of FCH developed after HCV recurrence in liver transplant recipients and treated with an interferon-free oral antiviral regimen (Table 1). A combination of SOF (polymerase inhibitor) and SMV (protease inhibitor) was used in these patients. Our study demonstrates that SOF-SMV combination is an effective regimen in OLT recipients with severe cholestatic hepatitis C recurrence. All of our patients achieved significant improvement of HCV viral load and they all had undetectable viral RNA PCR at week 6–8 of the treatment (Table 2). The HCV RNA remained undetectable throughout the treatment course; four of the five patients achieved SVR at week 12 after completion of the treatment. 

Generally, combination of SOF and SMV has excellent safety and side-effect profile, which makes it very attractive compared to interferon-based regimen. In a study of 167 patients treated with this combination, the COSMOS trial, the most common side-effects were fatigue (31%), headache (20%), and nausea (16%) [22]. SMV use was associated with photosensitivity and rash in other studies. Our first patient developed pruritus that was refractory to treatment and required frequent sessions of plasmapheresis. A self-limiting episode of acute pancreatitis developed in one patient which is difficult to attribute to the antiviral therapy. The second, fourth and fifth patients had very benign treatment courses with no side-effects recorded during the whole course of the treatment. The third patient was starting with multiple comorbid conditions. His course was complicated with hepatic artery thrombosis, and later developed sepsis and renal failure. 

Evidence for the use of SOF-SMV combination comes mainly from the COSMOS trial. In this trial, the overall SVR rates were 90% among the null-responders with no advanced liver disease and 94% among patients with advanced liver disease [22]. So far, clinical studies have observed no virologic breakthrough during therapy with SOF, which is consistent with the drug’s mechanism of action and high genetic barrier to resistance [17]. We found that aggressive treatment of post-OLT fibrosing cholestatic hepatitis C recurrence with SOF-SMV combination is recommended. Dedicated prospective studies are urgently needed to further evaluate treatment options in this patient population. 
